# Ultraweak Photon Emission as a Non-Invasive Health Assessment: A Systematic Review

**DOI:** 10.1371/journal.pone.0087401

**Published:** 2014-02-28

**Authors:** John A. Ives, Eduard P. A. van Wijk, Namuun Bat, Cindy Crawford, Avi Walter, Wayne B. Jonas, Roeland van Wijk, Jan van der Greef

**Affiliations:** 1 Samueli Institute, Alexandria, Virginia, United States of America; 2 Netherlands Metabolomics Centre, Division of Analytical Biosciences, Leiden Academic Centre for Drug Research, Leiden University, Leiden, The Netherlands; 3 Sino-Dutch Centre for Preventive and Personalized Medicine/Centre for Photonics of Living Systems, Leiden University, Leiden, The Netherlands; 4 Meluna Research, Amersfoort, The Netherlands; 5 Netherlands Organization for Applied Scientific Research, Zeist, The Netherlands; Glasgow University, United Kingdom

## Abstract

We conducted a systematic review (SR) of the peer reviewed scientific literature on ultraweak photon emissions (UPE) from humans. The question was: Can ultraweak photon emissions from humans be used as a non-invasive health assessment? A systematic search was conducted across eight relevant databases: PubMed/MEDLINE, BIOSIS, CINAHL, PSYCHINFO, All of Cochrane EBM databases, GIDEON, DoD Biomedical Research, and clinicaltrials.gov from database inception to October 2011. Of the 1315 studies captured by the search strategy, 56 met the inclusion criteria, out of which 1 was a RCT, 27 were CCT, and 28 were observational and descriptive studies. There were no systematic reviews/meta-analyses that fit the inclusion criteria. In this report, the authors provide an assessment of the quality of the RCT included; describe the characteristics of all the included studies, the outcomes assessed, and the effectiveness of photon emission as a potential health assessment tool. This report demonstrates that the peer reviewed literature on UPE and human UPE measurement in particular is surprisingly large. Most of the human UPE literature is of good to high quality based on our systematic evaluation. However, an evaluation tool for systematically evaluating this type of “bio-evaluation” methodology is not currently available and would be worth developing. Publications in the peer reviewed literature over the last 50 years demonstrate that the use of “off-the-shelf” technologies and well described methodologies for the detection of human photon emissions are being used on a regular basis in medical and research settings. The overall quality of this literature is good and the use of this approach for determining inflammatory and oxidative states of patients indicate the growing use and value of this approach as both a medical and research tool.

## Introduction

Bioluminescence is the process of production and emission of light by a living organism via chemiluminescence-based processes. Many examples are known in biology such as fireflies, Antarctic krill, fungi (for instance *Panellus stipticus*), various squid species, etc.

In fact, all cells produce some form of light emission, but most of this light is not visible to the unaided human eye. This photonic emission has characteristic wavelengths, duration, timing and patterns of flashes. These are features often associated with information and, while not proof in and of itself, it is reasonable to assume that these light emissions contain and carry information about the biological systems that produced it.

Ultraweak photon emission by living systems, sometimes called low level chemiluminescence, is the result of normal biochemical reactions in which electrons transition in and out of electronically excited states. Detection and identification of these excited states is easily achieved in well-defined chemical systems. However, the task becomes more problematic in complex biological systems, e.g., in studies of isolated cells, organs, or intact organisms.

The advent of new photon counting technologies in the early 1960’s provided the tools to demonstrate the existence of a ubiquitous low level luminescence in organisms. This provided convincing evidence that light from living organisms was not restricted to life forms having special organs containing enzyme systems such as luciferase/luciferin. This early work was primarily done in the USSR [1], [2] with a few exceptions [3]. Russian research was sometimes translated into English [4]. By that time it had been established in inorganic chemical systems that chemiluminescence could occur in oxidative reactions as a consequence of the recombination of oxygen-containing radicals [5], [6] and thus provided information about radical reactions and oxidation mechanisms. Outside the USSR, the existence of this radiation was neglected or considered a phenomenon that could be caused by external interferences of an unknown nature [7]. In the 1970’s, the existence of this photonic emission from living organisms was confirmed by research teams from Australia [8], Poland [9], Japan [10] and the USA [11]. Emissions are in the order of 10-10^4^ photons/s.cm^2^, and have been demonstrated in bacteria, yeast, whole animals and plants as well as cells and homogenates from organisms.

For yeasts (Saccharomyces cerivisiae and Schizosaccharomyces pombe) it was established that ultraweak photon emission was dependent on oxygenation and that spectral composition differed between the exponential growth and the stationary phase of the culture [8], [12], [13], [14]. Similar studies were later performed on the bacterium Escherischia coli [15], [16]. In mammalian systems UPE was studied at the organ, cellular and subcellular level. Ultraweak photon emission of rat liver was oxygen dependent, and increased by infusion of hydroperoxides. Spectral analysis indicated a predominance of red light-emitting species arising from the singlet oxygen dimole-emission peaks [11], [17]. Similar spectral data as well as oxygen dependency were obtained with isolated hepatocytes. Estimations of biochemical markers of lipid peroxidation combined with counts of photon emission led to the conclusion that UPE monitors the steady state concentration of singlet molecular oxygen [10], [11], [17], [18], [19]. Therefore, ultraweak photon emission can provide a useful tool to examine oxygen-dependent radical damage which affords an advantage over parameters (such as malondialdehyde levels) measuring accumulative effects.

Since the early 1980’s, it has been known that peroxidal lipid reactions are important components in the etiology of diabetes, liver and lung diseases, arteriosclerosis, ageing and cancer [20], [21], [22], [23], [24]. The production of oxygen radicals during the respiratory burst of phagocytic cell activity plays an essential role in bacterial killing and in regulating the processes of acute inflammation [25], [26]. In view of the damaging effects of oxygen radicals on tissues, it follows that anything causing abnormal activation of phagocytes has the potential to provoke a self-destructive response, most strikingly seen in autoimmune diseases [27]. This realization led to the idea that ultraweak photon emission determined in blood might be a general marker for health and could be used for diagnostic and clinical purposes [28], [29]. Early studies established the interdependence between various diseases and UPE intensity by measuring the differences between the luminescence of the blood of healthy and diseased human subjects. UPE of blood from patients with diabetes mellitus, carcinomas and hyperlipidemia showed higher emission levels than those of the samples from healthy people [28].

The development of a diagnostic assay based on ultraweak photon emission remained limited due to difficulties and requirements of ultra-high sensitive photon counting systems in the application to biomedical measurements [10], [28]. The use of chemiluminigenic probes - substances whose oxidation gives a high yield of electronically excited products – solved many of these issues by increasing the photon output by several orders of magnitude. For this purpose luminol at concentration 0.1–10 µM has been employed, for instance in assays of phagocytosis [25], . Lucigenin has also been sporadically used as a chemiluminigenic enhancer [26].

The next great challenge for the field of ultraweak photon emission detection (in relation to oxidative processes) was to provide images of these low signals in addition to physiological information. A two-dimensional photon counting imaging of a rat’s brain was technically achieved in 1999 [33], [34]. The equipment used in this first experiment consisted of a two-dimensional photon counting tube, a highly efficient lens system, and an electronic device to record time series of a photoelectron train with spatial information. Another application of two-dimensional imaging of these photon emissions has been in the field of cancer. In bladder cancer, transplanted into the feet of nude mice, the increase in photon emission in early developing cancer indicates the actively proliferating cancer before any detection of necrosis, haemorrhage, leukocyte infiltration or crusta formation [35]. Cancer imaging in mice transplanted with ovarian cancer cells utilizing a highly sensitive charge-coupled device (CCD) camera system has confirmed the increased UPE of tumours [36].

**Figure 1:**
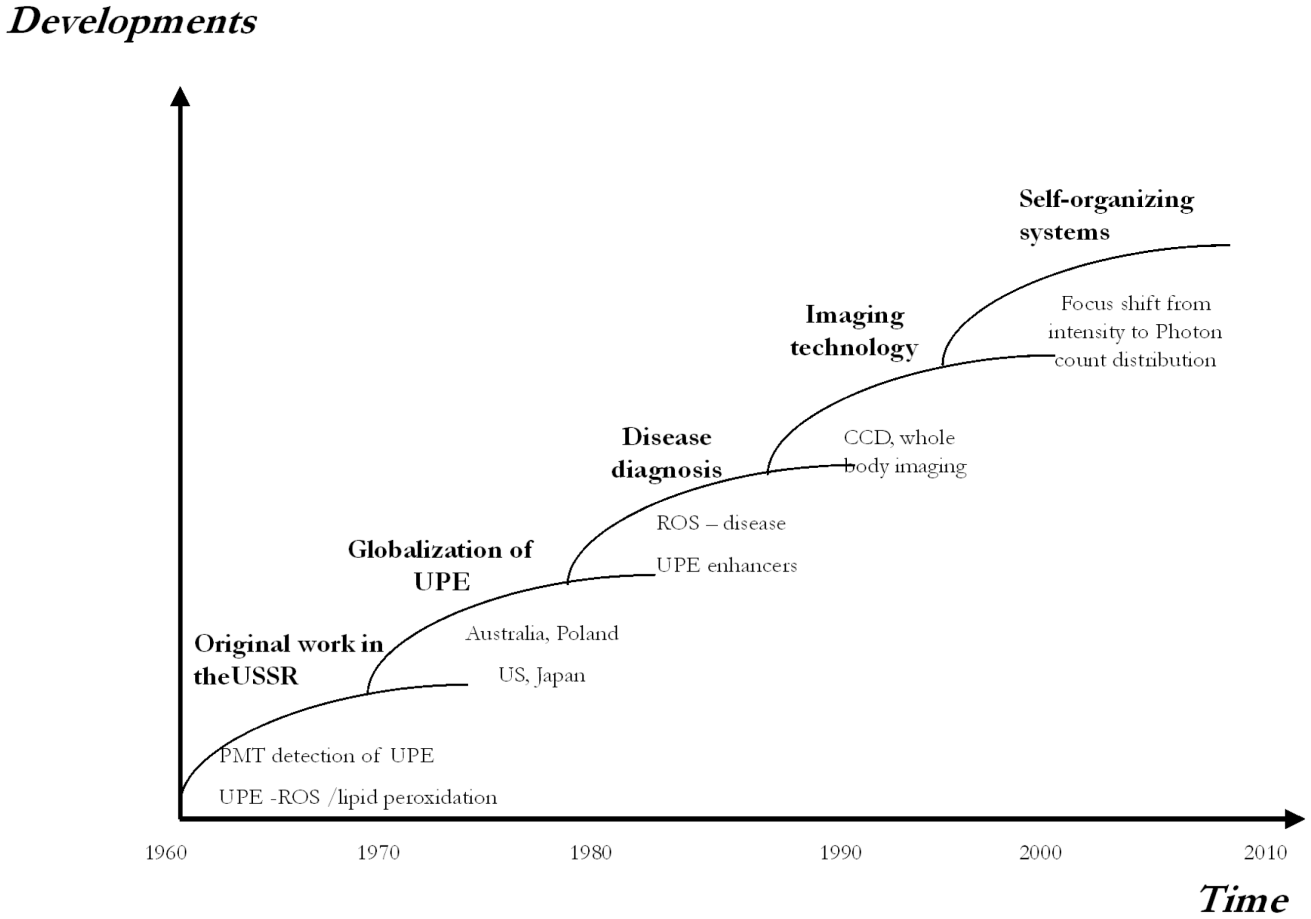
Major trends in UPE developments in the last 50 years. The historical development of this field can be subdivided in five main areas: (1) the initiation of research of UPE with photomultiplier tubes in USSR and its connection to radical oxygen species (ROS) and lipid peroxidation, (2) the recognition of UPE world wide and globalization of this research, (3) the use of UPE as a non-invasive diagnostic marker, (4) the extension of time measurement into spatial patterns, and (5) the use of photon count distributions (PCD) and statistics (PCS) (based on fluctuations in the number of photons in successive counting in contiguous measurement times) for detecting a ‘light language’ that is connected with the system’s organization of the living biological state.

The major trends over the last 50 years are schematically shown in Figure 1. Since the original observation of the effect, UPE detection methodology has matured to be uniquely positioned for not only providing insight into detailed biochemical processes but also provide a non-invasive tool for observing and even understanding system organisation.

Knowledge of the excitation state of biochemical reactions in organisms and isolated biological systems is of significant value for assessing basic biological function. In the case of whole organisms, a general and often specific assessment of health and wellness is possible with knowledge of the “reactive” or relative excitation state of intrinsic biochemical reactions. Therefore, it is possible, using detection and quantification of ultraweak photon emission, to non-invasively assess a person’s health, specifically the level of stress on biochemical and metabolic systems from the production of reactive oxygen species (ROS) as ROS are the principle source of these photons [5], [6], [22]. The 50 years of progress in this field has, thanks to advances in technologies for measuring ultraweak photon emissions, led to the emerging discipline of ‘human photon emission’.

To date, there have been no reviews of the literature to ascertain if ultraweak photon emission can be effectively used as a non-invasive health assessment tool. The purpose of this systematic review was to: 1) survey the literature on ultraweak photon emissions as they relate to human studies; 2) assess the quantity and quality of the research found; and 3) characterize the results by whether the study had an intervention or not, 4) what research model was studied, and 5) the potential value of using photons as a health assessment tool.

## Materials and Methods

### Search strategy

A systematic search was conducted across eight relevant databases: PubMed/MEDLINE, BIOSIS, CINAHL, PSYCHINFO, All of Cochrane EBM databases, GIDEON, DoD Biomedical Research, and clinicaltrials.gov from database inception to October 2011. The keywords related to photon were pre-identified by the team with guidance from subject matter experts. The final search terms decided upon and searched across databases were: photon, external bioenergy, spontaneous photon emission, ultraweak photon emission, ultraweak chemiluminescence, low level light emission, spontaneous chemiluminescence, ultraweak photons, ultra-weak photon emission, ultra-weak photons, ultra-weak chemiluminescence. See Appendix S1 for a full detail of the search in PubMed. Variations of this strategy were adapted for each unique database and are available upon request.

The search was limited to English language and human population only where databases allowed for these limitations. There was no limitation to study design in the initial search. Grey literature was searched by accessing and communicating with subject matter experts in the field and sharing their database collections as a cross reference to what the original search strategy retrieved as well as conducting basic internet searching across the keyword scheme. The reviewers also hand-searched bibliographies of all included studies to ensure that a comprehensive search strategy was used.

### Selection criteria

All study designs were included, however, for the purpose of the analysis, only the controlled clinical trial (CCT) and randomized control trial (RCT) study designs were considered. Studies were included in the analysis if they were: 1) in human populations, 2) describing the use of photons as a health assessment tool, 3) used in comparison with a placebo control or untreated control for the systematic review assessment, and 4) measuring ultraweak photon emission. Studies were excluded if they were categorized as: thought pieces, descriptive reviews, editorials, and all studies relating primarily to the physics of photons.

### Study selection

Three reviewers independently screened the titles and abstracts of all retrieved references according to the inclusion criteria presented above. All studies had to have some discussion concerning intrinsic light from live subjects in order to be considered for inclusion. Full text articles were pulled for all publications where a reviewer felt there was a potential for meeting inclusion criteria. At this point all study designs were included.

### Quality assessment and data extraction

The retrieved full-text articles were put into buckets of study designs by RCT, CCT, observational, descriptive, and mixed methods in order to assess the quantity and quality, and describe the characteristics of the individual included studies.

The RCT study design was assessed for quality according to the Scottish Intercollegiate Guidelines Network (SIGN 50) [37], a well-accepted and validated tool for assessing quality used widely in both complementary and alternative medicine (CAM) and conventional literature (see Table 1 for a full description of the SIGN 50 criteria). Because all of the rest of the studies reported here involved photons as an adjunctive assessment of the inflammatory state and not as the primary diagnostic assessment or necessarily including an intervention per se, the authors felt that the full SIGN criteria was not a good fit for evaluating the quality of the photonic portion of these studies. After examining the other quality assessment tools, the authors decided that none fit the photonic literature examined here. Because of this, the CCT study designs were only assessed across one of the criteria addressed in SIGN that fit. The other study designs (observational, descriptive and mixed methods) were only tallied as to how many fit the inclusion criteria laid out above.

**Table 1:** SIGN 50 Checklist for RCT Study Design.

Section 1: Internal validity	
Each item in Section 1 is to be evaluated using these criteria: 1) Well Covered, 2) Adequately Addressed, 3) Poorly Addressed, and 4) Not Applicable (NA) only for question 1.10.	
ITEM	DESCRIPTION
1.1	The study addresses appropriate and clearly focused question.
1.2	The assignment of subjects to treatment groups is randomized.
*1.3*	*An adequate concealment method is used.*
*1.4*	*Subjects and investigators are kept blind about allocation.*
*1.5*	*The treatment and control groups are similar at the start of the trial.*
1.6	The only difference between groups is the treatment under investigation.
1.7	All relevant outcomes are measured in a standard, valid and reliable way.
1.8	What percentage of subjects in each treatment arm dropped out before the study was completed?
1.9	All subjects are analyzed in the groups to which they were randomly allocated (intention to treat analysis).
1.10	Where the study is carried out at more than one site, results are comparable for all sites.

**Section 2: Overall Assessment**	
**How well was the study done to minimize bias? How valid is the study? Score options: ++, +, – based on following (modifications to SIGN criteria in italics):**	
**++**	**All or most** of the criteria have been fulfilled. Where they have not been fulfilled the conclusions of the study are thought very unlikely to alter. *An article receives this score if there are 0 criteria scored as poorly addressed.*
**+**	**Some** of the criteria have been fulfilled. Those criteria that have not been fulfilled or not adequately described are thought unlikely to alter the conclusions. *An article receives this score if 1-2 criteria are scored poorly addressed.*
**-**	**Some** of the criteria have been fulfilled. Those criteria that have not been fulfilled or not adequately described are thought unlikely to alter the conclusions. *An article receives this score if 1-2 criteria are scored poorly addressed.*

Note that only the RCT in Table 2 was assessed across all quality criteria in SIGN 50. For all CCT study designs in Tables 3 and 4, only criteria 1.1 appeared relevant to the focus of the study and therefore only scored for that criterion. See limitations section of the manuscript for a description of this challenge.

SIGN 50 Network: A Guideline Developer’s Handbook http://www.sign.ac.uk/guidelines/fulltext/50/checklist2.html

A data extraction form was created during the protocol development stage and tested by the team before use. This form captured details on the system of research, the objective of the study, the conditions and descriptions of the study population, the type of control, the specific outcomes and results related to photon emission and the authors’ main conclusions in order to provide a descriptive detail of the characteristics of the included studies.

All five reviewers were trained in the methodology for quality assessment and data extraction by the review manager (C.C.) and a rule book was created to assist in answering the questions objectively and consistently, which ensured reproducibility in the end, which was pilot tested among the team members. Forms were created in an online system offered through the Samueli Institute’s SharePoint cite to allow the reviewers to complete the data extraction. The 5 trained reviewers simultaneously extracted the data independently of each other (two reviewers assigned to each individual article) and all answers were tracked through this system. Any and all conflicts were resolved through discussions in team meetings. Once collected, all data was downloaded into an Excel table for each study and formatted in preparation for inclusion in Tables 2–4.

**Table 2:** Descriptive data for randomized controlled trials that have an intervention.

Citation	Objective of Study	Condition Group	Control	Results for outcome related to UPE	Author's Main Conclusion for Outcome of Interest	SIGN50 Score
Schutgens et al. 2009	To study the effect of plant adaptogens (Rhodiola rosea and ADAPT-232) on human UPE by measuring dorsal side of left and right hands.	10 (of 30) subjects with a mean age of 23.3, BMI of 21.7, and WHI of 0.87. 5 females and 5 males.	P	The Rhodiola group showed a statistically significant decrease in UPE (p = 0.027; pre = 2.94 and post = 1.72) in comparison with the placebo group (pre = 2.30 and post = 2.81). The ADAPT-232 and placebo groups had no significant changes (p = 0.199), although there was slight decrease in UPE in ADAPT-232 (pre = 2.39 and post = 1.82). *	Rhodiola rosea (SHR-5) reduced the experienced level of fatigue in comparison to the placebo group (p = 0.049). Both adaptogens (ADAPT-232 as well as Rhodiola rosea were able to reduce UPE from the dorsal side of both hands. Only Rhodiola rosea reduced UPE significantly compared to placebo (p = 0.027).	+

P  =  Placebo

**Table 3:** Descriptive data for clinical trials that have an intervention.

Citation	Objective of Study	Condition Group	Control	Results for outcome related to UPE	Author's Main Conclusion for Outcome of Interest	SIGN50 Criteria 1.1
*Whole Body/Skin*						
Hagens et al. 2008	To qualify and validate UPE measurement following ultraviolet (UV) excitation of porcine and human skin to assess the potency of topical antioxidants. Some subjects were treated with antioxidant standard formulations of 0.25%, 0.5%, or 1% vitamin C on both inner forearms twice daily for 3 consecutive days. Others received Glucosylrutin twice daily for 7 consecutive days on both inner forearms.	Vitamin C study: 20 female volunteers (age range of 18–65 years); Glucosylrutin study: 23 male and female subjects (age range of 22–60 years)	C	Topical vitamin C treatment reduced UVA-induced UPE (intensity in counts x 10^3^) in a dose dependent way. Reduction of the UPE signal, compared to control, was statistically significant for formulations containing 0.5% and 1% vitamin C. Topical treatment of skin with a formulation containing 0.25% a-glucosylrutin was also shown to significantly reduce UVA induced UPE. *****	UVA irradiation induces UPE, especially in deeper skin layers. Clinical data demonstrate UPE measurement following UV excitation is a reliable and valid method for the non-invasive measurement of antioxidant efficacy on skin. Thus, it can be assumed that reduction of UPE due to previous topical treatment with an antioxidant indicates a protection of living skin layers from pro-oxidative stress.	P
Raschke et al. 2004	To demonstrate the high in-vivo antioxidant capacity of 3% ascorbic acid.	27 (of 54) healthy female volunteers (age not reported)	P	The verum oil-in-water emulsion containing 3% ascorbic acid showed a significant reduction of UVA-triggered UPE compared to placebo. Vitamin C cream also exhibited a significantly stronger antioxidant potential (-75% change in UPE) than the verum containing 3% sodium ascorbyl phosphate (instead of ascorbic acid) in the same oil-in-water base (-51% change in UPE). The sodium ascorbyl phosphate emulsion showed no significant difference of UPE reduction compared to placebo (p = 20%) (-31% change in UPE). *****	Ascorbic acid is superior to sodium ascorbyl phosphate in an oil-in-water emulsion as a topical antioxidant for skin protection.	P
Jain 2010	To determine UPE decay characteristics of human skin and the modulatory influence of topically applied antioxidants. Six test areas were randomized on the back of the test subjects and measured.	12 healthy subjects with a mean age of 45.7+/−13.4 years (gender not reported)	C	UVA-induced UPE was characterized by two distinct decay phases: an initial 0–5 s burst approximately 80% of the complete signal with an inverse dose-response relationship, followed by a second decay phase showing a direct correlation. Antioxidant pretreatment caused a reduction in signal of about 50% during phase 1. The highest antioxidant efficacy (50%) was obtained with the smallest UVA dose of 126 mJ/cm2 (shortest irradiation time), whereas the difference in antioxidant efficacy obtained with 504 and 1008 mJ/cm2 of UVA was marginal(42 and 44%) *****	Sunscreens and antioxidants have different effects on cutaneous ROS generation. Sunscreens simply reduce the UVA intensity reaching the skin. As a result, cutaneous ROS generation does not depend on the irradiation time or UVA intensity, but on the applied UVA dose. Antioxidants do not just partly block UVA radiation, they also exert complex reactions with UVA-induced ROS, like resonance stabilization, and interact with other antioxidants or cutaneous enzymatic antioxidative systems as shown in this work by the modulation of the intersection point of decay curves at constant irradiation intensity. In summary, the data indicates that induced ultraweak photon emission of human skin is a sensitive, reliable, noninvasive method for studies of antioxidants or UVA filters.	A
Van Wijk 2010	To evaluate anti-oxidant activity of a specific topical OPCs formulation (anti-oxidant bioflavanoid) following UV exposure of the back of both left and right hands using the UPE assessment method. The authors hypothesized that the anti-oxidant effect of the OPCs cream would not only a) decrease skin UV-induced UPE but also b) protect the skin from sensitization to UV.	25 healthy female volunteers, age 19–68 years. Subjects with acne, eczema or hyper-pigmentation on the back of the hands were excluded. All subjects had Fitzpatrick skin type IV – VI.	U	The average level of spontaneous UPE from all subjects was 4.71+/−2.26 cps (min. 2.43 max.15.81 cps). Repeated left hand exposure to UV without treatment of OPCs cream demonstrated a steady increase in levels of UPE (statistically significant between recordings). However, topical OPCs cream applied to the right hand after UV resulted in a statistically significant decrease in UPE. *****	Results suggested a) that a reliable and valid protocol can be established to assess UV-induced oxidative stress in the skin, b) *in vivo* effects of topical antioxidants following exposure to UV can be demonstrated with non-invasive measurement of human UPE from skin and c) the topical OPCs cream investigated in this study can significantly reduce UV-induced UPE and protect skin from sensitization to UV.	W
Park 2009	To analyze the magneto-acupuncture stimuli effects on UPE of human hands.	45 healthy persons (29 men 16 women) 18–50 years of age. 8 subjects served as controls (Group 1), 4 subjects were treated with sham magnets (Group 2), and 33 subjects received treatment with magnets (Group 3).	U; P	UPE changes (counts per second) in control and sham groups were not significant. However, average and standard deviation UPE changes from the treatment group were evident in their palms. *	Average intensity and standard deviations for group 3 changed significantly compared with groups 1 and 2.	A
*Blood Cells*						
Holzer et al. 2010	To characterize neutrophil phenotype and function at close intervals; pre-, intra-, and postoperatively, in uneventful partial hepatectomies.	14 (of 19) non-cirrhotic subjects with liver tumors undergoing liver surgery.	U	Spontaneous oxygen radical generation by neutrophils (measured by lucigenin enhanced UPE in counts per minute/neutrophil) was low 24 h preoperatively and had no significant differences compared to healthy volunteers (n = 5). 15 min after the release of the Pringle maneuver, spontaneous oxygen radical production by neutrophils peaked (p<0.05 vs. baseline). Afterwards, spontaneous oxygen radical production by neutrophils decreased again. ***	Activation of neutrophils during liver surgery may be detrimental because an increase in adhesive properties together with an increase in spontaneous oxygen radical production may result in tissue accumulation of neutrophils and subsequent tissue damage, especially to the liver remnant. Spontaneous production of oxygen radicals peaked sharply 15 min after Pringle maneuver was opened. Formyl-methionyl-leucyl-phenlalanine(FMLP)-stimulated oxygen radical production was unchanged 15 min after Pringle maneuver was opened compared to baseline. Three hours after opening the Pringle maneuver, there was a decrease in FMLP-stimulated oxygen radical production by neutrophils (ns). Long-lasting effects of liver surgery demonstrated, at 48 and 120 h, FMLP-stimulated oxygen radical production exceeded baseline significantly.	A
Terpigorev et al. 2003	To study the morphology and functions of peripheral blood monocytes *in vitro*, their reactions to GC (glucocorticoid), and the correlation between cell sensitivity to GC and the results of glucocorticosteroid therapy in non-severe asthma patients.	42 (of 50) patients with persistent mild (n = 4) and moderate (n = 38) asthma (ages 16–69; 22 women 20 men). During the study, subjects stayed in the hospital under conditions free from contact with allergens, and received standard inhalation GC therapy in a daily dose of 1000 ug.	U	Initial parameters of baseline UPE in asthmatics and donors differed significantly (0.057+/−0.011 and 0.033+/−0.005, p<0.05). Baseline parameters of UPE were the same in patients with different efficiency of GC therapy, while after incubation of mononuclears with prednisolone in different concentration some differences between groups were revealed. UPE depression indexes revealed: the decrease of UPE parameters in group 2 (moderate GC responders) was more pronounced than in groups 1 (rapid GC responders) and 3 (non GC responders), which confirmed slight inhibitory effect of prednisolone on monocyte activity in vitro in group 2 patients. **	Signs of activation of circulating monocytes (hyper production of reactive oxygen forms) were observed in subjects with non-severe asthma. A significant correlation between the depression of monocyte activity by GC *in vitro* and the time of attaining clinical and functional remission during high-dose budesonide therapy was detected in steroid-sensitive subjects with non-severe asthma.	A
Tsai et al. 2004	To report that prophylactic high-dose L-arginine has a high capability of interacting with diabetic sera to generate harmful superoxide anions (O–2) as by-products which may subsequently propagate to form various types of other harmful free radicals.	10 (of 20) diabetic patients with a blood glucose level and HbA1c concentration higher than 200 mg/dl and 10%, respectively (age not specified).	U	When a fixed concentration of L-arginine was added to each of the diabetic sera, a higher amount of UPE (in counts/min) was generated, p<0.001. Conversely, L-arginine did not seem to elicit UPE when added to non-diabetic serum under similar conditions. ***	Data revealed the interaction between methylglyoxal glycation and *L*-arginine generated harmful O-2 as a by-product and subsequently could exacerbate the oxidative stress status of diabetic patients. For this reason, *L*-arginine should not be considered a preferable agent for blocking initial protein glycation by MG in diabetic patients.	A
*Blood Plasma*						
Hans 1997	To investigate the effect of total intravenous anesthesia (TIVA) maintained with a continuous propofol (PPF) infusion on the plasma antioxidant capacity (PAOC) in neurosurgical patients.	18 (11 women 7 men, mean age of 53.2+/−22.5 years) patients scheduled to undergo the placement of a cerebrospinal shunt for nontraumatic hydrocephalus.	C	Plasma Antioxidant Capacity (assessed by UPE) increased significantly during anesthesia in all but 3 patients (p<0.001). Plasma's capacity to inhibit lipid peroxidation increased from 39.8+/−2.8% to 44.7+/−2.4%. No significant correlation was observed between increased resistance to lipid peroxidation and blood propofol concentrations (r = 0.07, NS). *	Continuous PPF infusion increased plasma antioxidant activity in patients during TIVA. Although no correlation was observed with blood PPF concentrations, this effect could be caused by PPF. Ultimately, there is no definitive proof yet.	A
*Saliva*						
Goi 2007	To examine the response of oral peroxidase OPO reactivity to the Kraepelin test as a mental arithmetic stressor in smokers and non-smokers in addition to measuring: uric acid and IgA concentrations, flow rate, amylase activity as a salivary stress marker, thiocyanate level and UPE, which is indicative of salivary antioxidative and antibacterial abilities. The effect of smoking on the response of salivary peroxidase (SPO) and myeloperoxidase (MPO) activity to mental stress was also studied.	10 smokers (9 male 1 female) and 39 non-smokers (20 male 19 female) with mean ages 21.8+/−0.7 (range 21–23) and 21.3+/−1.1 (range 20–25).	U	Salivary UPE (measured by total number photons for 100 seconds after addition of H202) in the non-smoking group increased significantly just after the test, but changes in parameters were not significant (0 min before test = 13.02+-6.53 counts/100 sec, 0 min after test = 16.62+-7.72 counts/100 sec). UPE levels were significantly higher in smokers, compared to non-smokers, following the test (non-smokers 0 min after test = 16.62+/−7.72 counts/100 sec, smokers 0 min after test = 11.40+/−3.98 counts/100 sec) ****	Results of the present study show defensive components are stronger in non-smokers than smokers. The IgA concentration, Amylase activity, and UPE were higher for non-smokers than smokers regardless of the Kraepelin test (the amount of UPE reflects the ability to consume H2O2 as an antioxidant and produces OSCN-, and reflects the strength of the innate immune system).	A

SIGN50: How well does the study address an appropriate and clearly focused question? U  =  Untreated control; P  =  Placebo; C  =  Crossover; UPE  =  Ultraweak photon emission; cps  =  counts per second; CL  =  chemiluminescence; A  =  Adequately covered; W  =  Well covered; P  =  Poorly addressed; * No substance was used to amplify the ROS to photon reaction; ** Luminol was used to amplify the ROS to photon reaction; *** Lucigenin was used to amplify the ROS to photon reaction; **** Hydrogen peroxide in presence/absence of iron sulfate was used to amplify the ROS to photon reaction; ***** UVA was used to amplify the ROS to photon reaction

**Table 4:** Descriptive data for clinical trials that have no intervention.

Citation	Objective of the Study	Condition Group	Control	Results for outcome related to UPE	Author's Main Conclusion for Outcome of Interest	SIGN 50 Criteria 1.1
*Whole Body/Skin*						
Van Wijk et al. 2006	To systematically quantify the UPE emission of the anterior torso, head and neck plus the hands of long-term transcendental meditators (TM).	10 (of 20) male experienced practitioners of TM (mean age 50.4±4.3 yrs) who practiced no other meditations.	U	UPE intensity was lower in experienced TM practitioners; p = 0.03. TM practitioners had higher contributions of hand emissions and lower contributions of throat emissions to total emission compared to controls.*	Data support the hypothesis that free radical reactions, and thus UPE, can be influenced by TM.	A
Van Wijk et al. 2008	To record spontaneous UPE at 12 anatomic locations on subjects with long-term experience in transcendental meditation (TM), compared this with group that practiced other meditation techniques (OMT) and subjects with no meditation experience.	20 (of 60) experienced male TM practitioners (mean age of 51.3+/− 6.7 years) and 20 experienced male practitioners of OMT (mean age of 46.3+/−10.7 years).	U	Average overall UPE was lower by 27% in TM and 17% in OTM group, compared to controls. This was true overall and at each recorded anatomic location, indicating systematic differences between meditators and non-meditators; p = 0.0002. TM practitioners demonstrated lower emissions than OMT practitioners in 11 of 12 anatomical locations, indicating systematic group differences; p = 0.0032. *	Current data using noninvasive photon emission recordings suggest that, in addition to intensity and wavelength, the UPE of subjects may eventually be used to understand and delineate the state of mind-body integration (i.e. health) and the role of meditation programs in chronic disease.	W
Van Wijk 2008	To systematically quantify photon count distributions in subjects with or without long-term meditation experience at 12 different anatomic locations including upper frontal torso, head, neck, and hands.	60 healthy male subjects: 20 experienced TM practitioners (51.3+-6.7yrs), 20 experienced OMT practitioners (46.3+-10.7), and 20 control subjects with no meditation experience (43.4+-15.5).	U	Average overall UPE was lower by 30% in TM and 20% in OMT group, compared to controls. Signals emitted from forehead and both sides of left hand showed a greater decrease. *	A procedure was developed to analyze fluctuations of UPE by measuring the probability of emission and correcting for background noise. The values indicate that the quantum state of photons emitted by the subject could be a coherent state in those being investigated.	A
*Tissue*						
Grasso et al. 1992	To measure radiation emitted by samples of tumorous and normal human tissues coming from surgical operations.	16 (of 25) samples were tumorous human tissue taken from surgeries.	U	UPE was higher in tumor samples (average 300±90 photons/cm^2^ min) compared to normal samples (average 22 +/−6 photons/cm^2^ min). *	Results demonstrated that tumor samples have a greater UPE than normal tissue, allowing them to be clearly differentiated. More data is needed, but it is possible that UPE could represent a simple, non-invasive analytical tool for tumor diagnosis.	A
Keshavarzian et al. 1992	To determine whether excessive reactive oxygen metabolites (ROMs) are generated by inflamed colonic mucosa and to identify possible sources and types of ROMs. Mucosal ROMs were measured in rats and humans using a chemiluminesence probe.	7 (of 11) with documented ulcerative colitis (mean age of 35 years; 5 male and 2 female). All UC subjects had marked inflammation with crypt abscesses in their mucosal biopsy specimens.	U	UPE was significantly higher in colonic mucosal biopsy specimens from patients with acute ulcerative colitis (∼8000counts/min/mg) than normal mucosa (1000counts/min/mg); p<0.05. Adding catalase to the tissue suspension decreased UPE by the inflamed mucosa (∼5500counts/min/mg). **	Data indicates that excessive ROMs, and thus UPEs, are produced by inflamed colonic mucosa in both humans and rats, and may contribute to tissue injury.	A
*Blood Cells*						
Alexeyev et al. 1994	To study spontaneous and *Bacillus anthracis* induced luminol-dependent UPE of neutrophils in anthrax.	6 (of 11) subjects with suspected anthrax (5 males and 1 female). All exhibited high fever, fatigue, and multiple cutaneous painless ulcers located on their upper extremities.	U	UPE was lower in anthrax neutrophils than in healthy blood donors; p<0.05. *B. anthracis* did not induce chemiluminescence in anthrax neutrophils. However, *B. anthracis* did stimulate ∼4-fold increase in UPE in controls; p<0.01.**	This study demonstrated that spontaneous and *B. anthracis* induced chemiluminescence is impaired in anthrax, whereas the functional capacity of antibodies seems to be unaffected.	A
Hammann et al. 1987	To study the possible changes in the T-cell population during increased UPE. The chemiluminescence activity (CL-A) and the percentage of OKT3, OKT4 and OKT8 positive peripheral blood cells were serially examined.	8 (out of 12) Multiple Sclerosis (MS) patients (mean age 30 +/− 11.2 years; 7 females and 1 male).	U	When the OKT values were obtained from MS patients in phases of increased CL-A (clinical remission) the percentage of OKT3-positive cells was reduced (p = 0.014), OKT4-positive cells increased (p = 0.014), and there were no significant changes in OKT8-positive cells (p = 0.171) compared to controls. **	When MS patients start to improve clinically, the spontaneous CL-A of their peripheral blood monocytes significantly increases. Thus, there are changes in the number of OKT3- and OKT4- positive cells which occur together with an increased CL-A.	A
Ho 2000	To investigate the relation between ankylosing spondylitis (AS) and the oxidative metabolism of phagocytes in whole blood.	24 (of 45) male patients with Ankylosing Spondylitis (AS) (mean age 35.6, range 19–54 years). All patients were HLA-B27 positive and had not taken NSAIDs in the past 2 weeks.	U	Compared with healthy subjects, AS subjects had a higher intensity of lucigenin enhanced UPE, with or without fMLP or PMA stimulation. The rate of superoxide anion radical production (counts/10s/105 phagocytes) in patients with AS was significantly higher than in healthy subjects both when their blood was in the resting or stimulated by fMLP or PMA, with average increases of 8.8, 4.1, and 4.5 times, respectively (p<0.01). ***	Superoxide anion radical production and lucigen enhanced UPE in the blood of patients with AS is increased in both resting and stimulated states. Primed phagocytes may be one of the causative factors in the pathogenesis of AS, but further research is required.	A
Koval'chuk et al. 1998	To study the effect of naturally occurring cytokines on UPE of neutrophils from bronchoalveolar lavage and peripheral blood in chronic bronchitis patients.	20 subjects with chronic bronchitis (aged 23–65; 8 male and 12 female). 8 males had obtrusive bronchitis, 4 had lingering pneumonia and 2 had broncoectasia.	U	Unstimulated UPE was higher in the bronchoalveolar than in peripheral blood neutrophils: 11.2+/−1.21 vs. 4.16+/− 1.04 mV (p<0.05). The maximum amplitude of cytokine induced luminol-dependent UPE was much lower (up to 10-fold) than that of peripheral blood neutrophils: 61+/−12.7 and 170.9+/−24.7 mV, respectively (p<0.05). Thus, the index of luminol-dependent UPE stimulation was much lower in bronchoalveolar neutrophils in comparison with that in peripheral blood neutrophils. **	It can be concluded 1) that in chronic nonspecific lung diseases the generation of the active oxygen radicals by bronchoalveolar neutrophils is changed compared to peripheral blood neutrophils and 2) naturally occurring cytokines stimulate the production of active oxygen forms by peripheral blood neutrophils in patients with chronic nonspecific pulmonary diseases.	A
Safronova et al. 2003	To study the generation of active oxygen forms by blood granulocytes in subjects with a history of habitual abortions.	23 (of 35) nonpregnant women with a history of abortions (2–3 spontaneous during I trimester and undeveloped pregnancies) (age 21–35 years)	U	Comparison of blood UPE parameters showed that in women with a history of spontaneous abortions, the basal UPE level and the maximum amplitude of response to opsonized zymosan were notably higher, and the activation index significantly lower (p<0.001). Basal level of isolated granulocyte spontaneous UPE was significantly higher in patients with a history of miscarriages, while the maximum amplitude of responses to chemotactic peptide was lower in this group than in controls (p<0.01). **	Results show oxidative stress and poor cytotoxic functions of granulocytes in women with a histsory of spontaneous abortions, which can be due to specific features of regulation of oxidase activity by tyrosine protein kinases and protein phosphatases and by p38 MAPK. These data give us grounds to consider that signaling from the chemotactic peptide receptor to NADPH oxidase, responsible for AOF generation, is changed in granulocytes from women who have had abortions, and can lead to changes in the inflammatory process.	A
Zimmermann et al. 1999	To determine whether neutrophil function is impaired in patients with severe pneumonia, the 2 main partial functions: exocytosis and oxidative response (ROS production) was studied.	21 (of 31) patients with severe pneumonia (mean age 62; 13 males and 8 females). 11 were mechanically ventilated and 10 breathed spontaneously. 14 were hospital-acquired and 7 had community-acquired pneumonia.	U	UPE was higher in pneumonia patients (13.6×10^5^ cpm) than in controls (5.5×10^5^ cpm). Both basal and PMA-stimulated ROS production were increased in patients compared to controls.**	Patients with severe pneumonia had significantly impaired exocytosis of blood neutrophils and increased oxidative response.	A
*Other Cells*						
Clark et al. 1988	To study the pulmonary cellular response (alveolar macrophages) of fire survivors with cutaneous burns, smoke inhalation, or combined injury.	42 (of 60) fire victims with acute lung injury after burns and smoke inhalation. Of the fire victims: 10 had clinical and biochemical evidence of smoke inhalation but no cutaneous burns, 15 had cutaneous burns only, and 17 suffered both smoke inhalation and cutaneous burns.	U	Spontaneous UPE and stimulated UPE, were similar in cells from patients with cutaneous burns and controls. Cells from patients with only smoke inhalation showed similar UPE compared to controls, but with a significant increased response to stimulation (p<0.05). Patients with combined injuries had a significant increase in spontaneous and stimulated UPE compared to controls (p<0.05). Stimulated response in the combined injuries group was significantly less than the smoke inhalation group (p<0.05). *	The size of the alveolar cellular response to smoke and cutaneous burns suggests that lung damage follows from excess release of inflammatory mediators, exhaustion of the reserve of mature phagocytes and consequent reduced ability to fight bacteria, or both.	A
*Blood Plasma*						
Calabrese et al. 1998	To study the content of sulfhydryl groups and products of lipid peroxidation, including UPE and liposoluble fluorescence in cerebrospinal fluid (CSF) and plasma of Multiple Sclerosis patients.	15 (of 30) adults with primary diagnosis of Multiple Sclerosis (MS). (Age and gender not reported)	U	Plasma and CSF levels of UPE (luminescence units/ml) were higher in MS patients (plasma 0.18+/−0.02; CSF 0.16+/−0.01) than in controls (plasma 0.11+/−0.009; CSF 0.09+/−0.009); p<0.05. Plasma and CSF levels of stress-induced UPE were not significantly different in MS patients compared to controls. Post-stress/spontaneous UPE ratio, indicative for susceptibility of oxidative stress, for CSF and plasma was lower in MS patients than in control group, p<0.05. *	There was a decrease in sulfhydryl groups and increased content of products of lipid peroxidation, such as UPE and liposoluble fluoreschece, which were found to be higher in the CSF and plasma of MS patients, pointing out the role of oxidative stress in the pathogenesis of MS.	A
Yoda et al 1985	To study the effect of smoking on UPE by looking at blood plasma in subjects.	27 (of 56) male smokers (mean age 47.2+/−7.1).	U	Plasma samples from smokers produced higher levels of UPE (125.2+/−36.9 counts/10 sec), approximately twice those of nonsmokers (55.7+/−12.6 exsmokers and 53.5+/−14.2 neversmokers); p<0.001. *	Effects of smoking on UPE are significant, but appear to be short term. Further study is needed to determine if the assumption that plasma UPE of smokers' blood might be related to carcinogenic action of cigarette smoke and to cigarette smoking-associated disorders through the generations of free radicals and active oxygens.	W
Agatsuma 1992	To study the potential relationship of a UPE substance (with a peak at 430 nm) and the blue fluorescent compounds observed on the HPLC-gel chromatography of the plasma of hemodialysis patients. Absorption, fluorescence, and elution patterns were studied.	Hemodialysis patients (Age and gender not reported)	U	The only difference between subjects was a small emission peak at 430 nm in hemodialysis patients, upon introduction of H2O2. The same amount of H2O2 added to plasma in the presence of iron sulfate resulted in the hemodialysis plasma showing a clear emission peak at 430 nm, with a second small peak near 680 nm. Normal subjects, in contrast, displayed a broad emission peak at 680 nm. (1104 +/−261 counts/s for healthy subjects; 8398+/−7011 counts/s for hemodialysis patients). ****	UPE intensity at 430 nm induced by the attack of hydroxyl radicals is specific for the plasma of hemodialysis patients, the characteristic UPE factor being isolated specifically in the low-molecular-mass fractions of the hemodialysis patient’s plasma upon HPLC-gel chromatography.	A
*Urine*						
Barbieri et al. 1999	To study the antioxidant defenses of varicocele patients both at the local (seminal plasma) and systemic (blood plasma) levels.	50 (of 61) semen samples from male untreated varicocele patients (17–39 years). The 50 subjects were further divided into 35 Normozoospermic, 7 Asthenozoospermic, and 8 Ligoasthenospermic.	U	Compared to healthy subjects (1800+/−800), urinary UPE (counts per minute/milligram of creatinine) was higher in the 3 study groups: Normozoospermic (5430+/−4700); Asthenozoospermic (5400+/−2000); Oligoasthenospermic (3600+/−1400); p<0.0001.*	Varicocele-associated oxidative stress was seen both at the local and systemic levels. Data show that varicocele patients, even those with normal spermiograms, present considerably higher urinary UPE than controls. Also, it is interesting to note that the range of values in patients is considerably wider, which could indicate a particularly strong oxidative stress. In such cases antioxidant therapies may be beneficial.	A
*Gisler et al. 1982*	*To study variables that might affect urinary UPE, and try to standardize these procedures.*	*9 (of 18) cancer patients, all nonsmokers*	*U*	Urinary UPE (counts/6 sec x 10^−3^) was higher in smokers than in non-smokers and very low in cancer patients. Temperature increase showed increased UPE, which decreased as the urine cooled. At basic pH = 10, UPE was higher than at acidic pH = 1 and at native pH = 5.2. Ingestion of ascorbic acid decreased UPE. Smokers consistently had higher UPE than the nonsmokers. Cancer subjects (4.46+/−2.18 counts/6 sec x 10^−3^) had lower UPE than normal subjects (11.39+/−5.25 counts/6 sec x 10^−3^) at pH = 1 and 16C. At native pH and 16C, normal subjects = 13.61+/−8.17, cancer = 5.99+/−4.98. At pH = 10 and 16C, normal = 364.6+/−132.3, cancer = 221.3+/−44.5. The respective results are also given for 58C in table 5. *	*The different protein pattern as well as UPE after filtration and from total urine, indicate that urinary UPE may have a potential for cancer detection in the initial stages. Spontaneous UPE in the urine could serve as a cancer marker.*	*A*

SIGN50: How well does the study address an appropriate and clearly focused question? U  =  Untreated control; P  =  Placebo; C  =  Crossover; UPE  =  Ultraweak photon emission; cps  =  counts per second; CL  =  chemiluminescence; A  =  Adequately covered; W  =  Well covered; P  =  Poorly addressed; * No substance was used to amplify the ROS to photon reaction; ** Luminol was used to amplify the ROS to photon reaction; *** Lucigenin was used to amplify the ROS to photon reaction; **** Hydrogen peroxide in presence/absence of iron sulfate was used to amplify the ROS to photon reaction; ***** UVA was used to amplify the ROS to photon reaction

All included studies were then characterized as to whether there was an intervention and what system of research they fell into (whole body, cells [blood cells, other cells], fluids [blood plasma, urine, cerebral fluid, saliva], and other [tissue]). These categories were chosen not only because the articles naturally broke into these categories but also they make intrinsic bio-medical sense.

The effect size was not calculated due to the heterogeneity of the trials identified during the review phase. All assessments were based on information provided in the published manuscripts that met the inclusion criteria.

## Results

**Figure 2:**
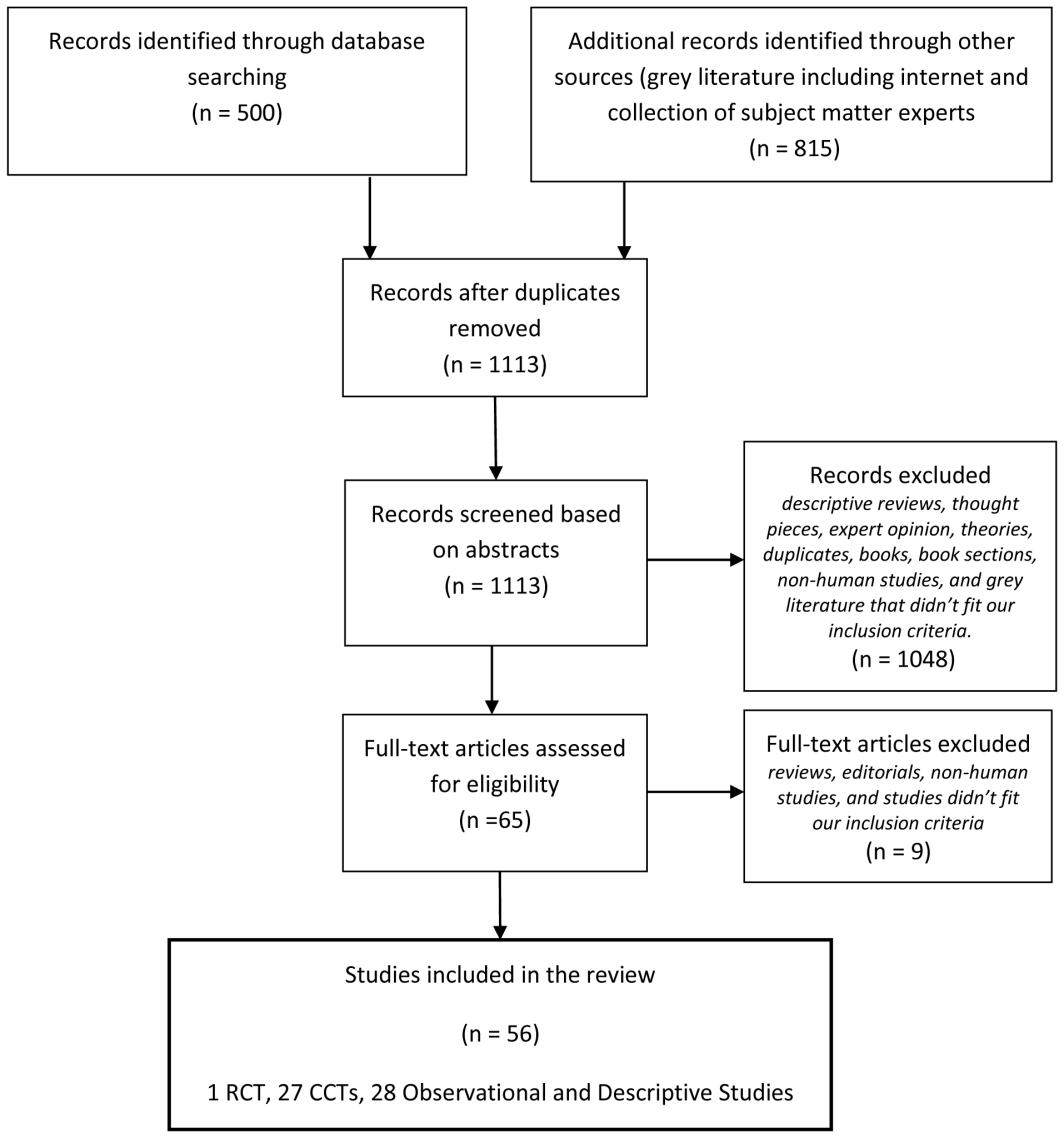
Flow chart. There were a total of 1315 records identified through the literature search, out of which 1113 were screened based on abstracts after removal of duplicates. The first level of screening based on abstracts resulted in exclusion of 1048 records (descriptive reviews, editorials, theories, books, book sections, non-human studies, and grey literature that didn’t fit our inclusion criteria), and the second level of screening based on full-text resulted in exclusion of 9 more additional records. Therefore, a total of 56 studies (1 randomized controlled clinical trial, 27 controlled clinical trials, and 28 observational/descriptive studies) were included in this review.

Of the 1315 studies captured by our search strategy, 56 met the inclusion criteria, out of which 1 was a RCT, 27 were CCTs, and 28 were observational and descriptive studies. There were no systematic reviews/meta-analyses that fit our inclusion criteria. See Figure 2 for a graphical depiction of the study selection and elimination process throughout the review phases. In this report, the authors provide an assessment of the quality of the RCT included; describe the characteristics of all the included studies, the outcomes assessed, and the effectiveness of photon emission as a potential health assessment tool. The RCT and CCT study designs are presented descriptively in Tables 2–4. Other formal study designs included are narratively summarized below and will be reported on in more detail in future publications.

### Quality assessment of RCT

Of the 56 included studies, only one was categorized as a RCT. In this study, the effect of plant adaptogens on human ultraweak photon emission was determined by providing subjects with supplements of *Rhodiola rosea*, placebo pills, or ADAPT-32 (mix of adaptogens) for one week, and measuring the ultraweak photon emission from the dorsal side of their hands. The results show a significant decrease in photon emission (p = 0.027), as well as in reported fatigue levels (p = 0.049) in the *Rhodiola* group compared to the placebo group. However, this work would have been better with the groups approximately matched for age. This RCT received an overall quality score of (+) according to the SIGN 50 criteria. The authors did not provide a full description of the randomization process and allocation concealment methodology in the published report. Although they did not address these important components, because of the strength in the other criteria assessed the conclusions of the study are thought unlikely to be altered by the inclusion of this missing information. We also believe there is a low level of bias being introduced to this study. Further studies employing an RCT design are needed to determine the value of photon emission measurement as a tool for assessing the impact of supplement use as well as other interventions meant to improve health. See Table 2 for further details of the study [38].

### Controlled clinical trials

Of the 56 included studies, 27 were characterized as CCT study design, out of which 17 studies involved no intervention (Table 4) while the remaining 11 studies had an intervention (Table 3). It is interesting to note that most interventional studies came up in the literature post-2003 with the exception of Hans 1997 [39], whereas all of the research in the last two decades of the 1900’s (1982–2003) involved no intervention. In the Hans study, the effect of total intravenous anesthesia on the plasma antioxidant capacity of neurosurgical patients during a cerebrospinal shunt was studied. It was a first of its kind to measure ultraweak chemiluminescence of patients’ plasma before and after an intervention. All the other research was focused on assessing and comparing the state of inflammation among different groups for future diagnosis purposes. These studies were grouped by their system of research. The most commonly studied system of research was blood cells (9) [40], [41], [42], [43], [44], [45], [46], [47], [48] followed by whole body (8) [49], [50], [51], [52], [53], [54], [55], [56], blood plasma (4) [39], [57], [58], [59], tissues (2) [60], [61], urine (2) [62], [63], saliva (1) [64], and other cells (1) [65].

The inflammatory states studied in the various publications included respiratory inflammations such as anthrax, chronic bronchitis, pneumonia, lung injury, and smoking as well as a number of other conditions such as, ulcerative colitis, multiple sclerosis, cancer, ankylosing spondylitis, hemodialysis, and varicocele. There were three studies [53], [54], [56] that looked at the ultraweak photon emission of experienced meditation practitioners compared to practitioners with no experience. All three studies found decreased photon emission in the experienced meditation groups compared to the control groups.

### Observational and descriptive studies

Of the 56 included studies, 28 were characterized as observational and descriptive studies, out of which 20 studies used whole body[56], [66], [67], [68], [69], [70], [71], [72], [73], [74], [75], [76], [77], [78], [79], [80], [81], [82], [83], [84], 3 blood cells[85], [86], [87], 3 saliva[64], [88], [89], 1 blood plasma[90], and 1 breath[91] as systems of research. The details of these studies will be reported on in future publications.

## Discussion

### Main findings

Publications in the peer reviewed literature over the last 50 years clearly demonstrate that the use of “off-the-shelf” technologies and well described methodologies for the detection of human photon emissions are being used on a regular basis in medical and research settings. Our search turned up only one RCT. While of good quality (SIGN score of “+”), clearly this field needs more RCTs to truly test the use of UPEs as a diagnostic tool. The overall quality of this literature is good and the use of this approach in medical oncology settings for determining inflammatory and oxidative states of patients indicate the growing use and value of this approach.

### Strength and limitations

Because of this system of research, there is no quality rating tool available in the literature to accurately assess the quality of UPE research. This type of research is neither diagnostic as defined in some of the quality tools for diagnostic studies nor do the studies we found consist of clinical trials with clearly defined intervention and control. SIGN 50 Diagnostic Tool for assessing quality looks at whether the results reflect the accuracy of the diagnostic test being evaluated. This is not what these groups of studies did as they were looking at photons as an adjunctive assessment of the inflammatory state and not as the primary diagnostic assessment, or including an intervention necessarily which made finding a tool to fit these designs of studies very challenging [37]. Other tools were explored and they too did not fit within the scope of the research examined in this review. The limitation of this systematic review is that we are not able to fully comment on the overall quality of these studies captured in this review without this tool in place. Since this research seems promising, it would be worthwhile to determine how best to assess quality of this system of research for future reviews. We were however able to document the quantity of the literature that exists in a rigorous, systematic way in this area and describe the literature as it exists today which is beneficial to understanding how it could be used as a health assessment tool and what has been done to date using this method. Since this research seems promising, it would be worthwhile to determine how best to assess quality of this system of research for future reviews. At this point, provided this limitation, the authors can only say subjectively that the quality is “good” for the CCT study designs evaluated.

### Implications for research

Technological advances and improved understanding of the underlying biology have combined to enable the measurement of human photons in a rapid and reliable manner. The technology has advanced sufficiently to allow for non-invasive whole body measurements of these relatively rare photons and there is some evidence that measuring from the hands is sufficient [92]. This has the potential to provide both the researcher and the diagnostician with a powerful tool to look within the body, in real-time, at the fundamental biochemistry and production of ROS and correspondingly the REDOX state of the metabolism [93]. While the production of ROS is an unavoidable by-product of metabolism [94], when it is not properly managed by the body or when it is excessive as under certain kinds of stress [95], [96] it can lead to a number of pathologies.

As ROS have been shown to be associated with a number of disease states it is clearly of use to assess their presence and prevalence. Therefore, assessing ROS levels may be useful for tracking the impact of various interventions, behaviors or medications. Doing so in a non-invasive and easy way is of great import.

## Conclusion

Our study demonstrates that the peer reviewed literature around UPE and human UPE measurement in particular is surprisingly large. Most of the human UPE literature is of good to high quality based on our systematic evaluation. However, an evaluation tool for systematically evaluating this type of “bio-evaluation” methodology is not currently available and would be worth developing. The authors applied their experience in performing systematic reviews to perform this evaluation from the available tools.

In addition, the field of human photon detection and the technology for measuring same has matured to the point where RCTs should be performed. In this way, it will be possible to determine if this approach can deliver a tool for non-invasively evaluating the specific inflammatory state of the individual while simultaneously providing a general measure of overall health. Finally, detection of human photon emissions has the potential for broad applicability to human health and disease monitoring at the whole systems level.

## Supporting Information

Checklist S1 **PRISMA 2009 Checklist.** PRISMA checklist contains the preferred reporting items for Systematic Reviews and Meta-Analyses. The section of this article where each item is addressed is provided here.
(DOC)

Appendix S1 **Pubmed Search Details.** The search terms and limits used in Pubmed, and the dates the search was conducted is provided here.
(DOCX)
