# Supplementary material for: Ultraweak Photon Emission as a Non-Invasive Health Assessment: A Systematic Review
Source: PLoS One. 2014 Feb 28;9(2):e87401. doi: 10.1371/journal.pone.0087401 (PMC3938423; doi:10.1371/journal.pone.0087401)
Supplement: Appendix S1 — Pubmed Search Details. The search terms and limits used in Pubmed, and the dates the search was conducted is provided here. (DOCX) [file pone.0087401.s002.docx]

**Appendix S1. Pubmed Search Details**

**Search Terms used in Pubmed:** biophoton* OR "external bioenergy" OR "spontaneous photon emission" OR "ultraweak photon emission" OR "ultraweak chemiluminescence" OR "low level light emission" OR "spontaneous chemiluminescence" OR “ultraweak photons" OR “ultra-weak photon emission" OR "ultra-weak photons" OR “ultra-weak chemiluminescence" OR “ultra weak photon emission” OR “ultra weak photons” OR “ultra weak chemiluminescence”

**Limits:** Clinical Trial, Meta-Analysis, Randomized Controlled Trial, Review, Case Reports, Clinical Trial, Phase I, Clinical Trial, Phase II, Clinical Trial, Phase III, Clinical Trial, Phase IV, Comparative Study, Controlled Clinical Trial, Evaluation Studies, In Vitro, Multicenter Study, English

Search was conducted in Pubmed on October 10, 2011.
